# Diversity of obligate ectoparasites and parasitism patterns in wild birds of the Balearic Islands: new chewing lice records for Spain

**DOI:** 10.1017/S0031182025101194

**Published:** 2026-02

**Authors:** Rafael Gutiérrez López, Mikel Alexander González, Julia López-Mercadal, Raul Escandell, Oscar García-Febrero, Rafel Triay, E. Coll, Miriam García, Ivan Bernal, Carlos Talabante, María L. Moraza, Miguel Angel Miranda, Carlos Barceló

**Affiliations:** 1 Centro Nacional de Microbiología, Instituto de Salud Carlos III (CNM-ISCIII), Madrid, Spain; 2 CIBER de Enfermedades Infecciosas (CIBERINFEC), Madrid, 28029, Spain; 3 Departamento de Soluciones ambientales y Entomología, Athisa Medio Ambiente - Grupo SASTI, Granada, Spain; 4 Applied zoology and animal conservation group, University of the Balearic Islands, Palma, Spain; 5 Sociedad Ornitológica de Menorca (SOM). Es Castell, Balearic Islands, Spain; 6 COFIB (Conselleria d’Agricultura, Pesca i Medi Natural, Govern de les Illes Balears). Ciutadella, Menorca, Balearic Islands, Spain; 7 Grupo de anillamiento Álula, Madrid, Spain; 8 Departamento de Control de Vectores, Madrid Salud, City council, Madrid, Spain; 9 Departamento de Ciencias de la Vida, Universidad de Alcalá, Madrid, Spain; 10 Grupo de anillamiento Álula, Grupo de anillamiento Álula, Madrid, Spain; 11 Institute of Biodiversity and Environment BIOMA, University of Navarra, Pamplona, Spain; 12 Laboratory of Zoology, University of the Balearic Islands, Palma de Mallorca, Spain; 13 Instituto de Investigaciones Agroambientales y de Economía del Agua (INAGEA), University of the Balearic Islands, Spain

**Keywords:** bird migration, feather mites, lice, parasites, ticks

## Abstract

Ectoparasites are commonly found on wild birds and might play an important role as vectors of pathogens. The Balearic Islands archipelago (Spain) is an ecological hotspot for wild birds due to its geographical location and habitat diversity. Although the avian fauna of the archipelago is well studied, little information is available regarding the ectoparasites infesting its wild bird populations. This study aimed to identify the diversity of ectoparasites (chewing lice, louse flies and ticks) and feather mites on wild birds in several locations on Menorca Island, as well as to assess the prevalence based on the migration status and season. Our research revealed that ten of the 13 species of chewing lice collected in this study are reported here for the first time in the Balearic Islands, including two that are also new records for Spain. We did not find statistically significant differences in the prevalence of ectoparasites or feather mites between sedentary and migratory birds. Likewise, no significant differences were observed in feather mite prevalence among migratory birds from Illa de s’Aire between prenuptial and postnuptial migrations. This study provides valuable insights into the prevalence and diversity of ectoparasites, shedding light on their potential role as vectors for avian pathogens. Further research is needed to explore the pathogens these ectoparasites may carry and transmit, contributing to a better understanding of the epidemiology of avian diseases in Menorca.

## Graphical abstract

**Figure:**
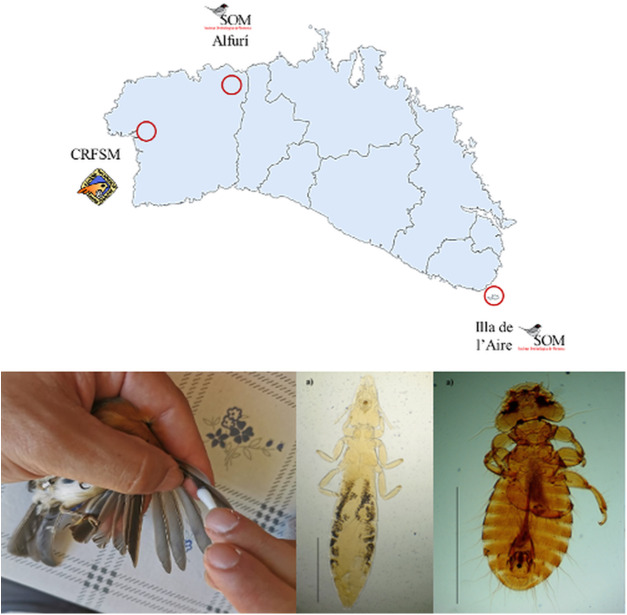

## Introduction

Wild birds are significantly affected by permanent ectoparasites, including mites, ticks, louse flies, fleas and chewing lice (Calvete *et al.,* 2003; Sychra *et al.,* 2011; Oslejskova *et al.,* 2020). These parasites may directly affect bird condition and fitness by causing stress and injuries that compromise its well-being, such as body condition, anaemia, reduction of flight ability, thermoregulation issues and behaviour changes (Lehmann, 1993; Martínez-de La Puente *et al.,* 2011; Defaye *et al.,* 2023; Espí *et al.,* 2023). Such effects have lasting implications for the growth and reproductive success, especially on passerine birds (Fitze *et al.,* 2004), but also on other avian groups like prey birds (Lesko and Smallwood 2012; Reed *et al.,* 2012). In addition to their ecological relevance, ectoparasites play a central role in shaping host behaviour, life history, and population dynamics (Poulin, 2007).

Despite this importance, systematic surveys of avian ectoparasites remain scarce, particularly in southern Europe and island ecosystems. In the Iberian Peninsula, most studies have focused on specific host groups or parasite taxa (e.g. Moreno-Rueda and Hoi 2012; Tomás *et al.,* 2016), leaving significant knowledge gaps on broader host–parasite associations. In the Balearic Islands, no comprehensive studies have yet described the diversity or prevalence of ectoparasites in wild birds. In this sense, Menorca is an island with the UNESCO status of Reserve of the Biosphere since 1993 (UNESCO 1993). The island belongs to the Balearic Island archipelago (Spain) located in the western Mediterranean basin, and has typical Mediterranean climate following the Köppen climate classification, by warm temperatures in summer, with an average annual temperature of 17.2 ºC and average annual precipitation of 545 mm (AEMET 2022). Its landscape comprises diverse natural habitats, ranging from wetlands and coastal areas to Mediterranean forests, which sustain rich avian biodiversity. The island is strategically located along East Atlantic flyway and serves as a key stopover site for migratory birds traveling between northern Europe and Africa. This convergence of migratory and resident species within the same habitats creates opportunities for ectoparasite spillover, co-infestation, and the establishment of novel host–parasite associations (Altizer *et al.,* 2011; Tomás *et al.,* 2016). Such conditions make Menorca a natural laboratory for studying ectoparasite dynamics in insular ecosystems, where local ecological constraints and the mixing of host populations can shape parasite prevalence and community composition in unique ways (Poulin, 2007; Krasnov *et al.*, 2012). Therefore, establishing baseline data in such region is essential for understanding parasite biogeography, assessing host–parasite network structure, and providing a reference for future ecological and epidemiological research (Krasnov *et al.*, 2012; Bush and Clayton 2018).

An additional question of ecological interest is whether ectoparasite prevalence differs between migratory and sedentary species. Migratory birds may be exposed to more parasites through contact with multiple host populations along their flyways, potentially increasing prevalence (Altizer *et al.,* 2011). Conversely, sedentary birds may accumulate higher infestations due to continuous exposure within local habitats, acting as ‘sitting ducks’ for parasite transmission (Poulin, 2007). Previous studies have reported mixed results, for instance some authors described higher prevalence in migrant birds (Sychra *et al.,* 2011), while others observed similar or even higher prevalence in resident species (Sychra *et al.,* 2008; Tomás *et al.,* 2016; Gustafsson *et al.,* 2019). These inconsistencies suggest that host traits, ecology, and environmental conditions strongly modulate parasite dynamics.

An example of those ectoparasites is the feather mites (Acari: Astigmata), these are specialized arthropods that inhabit bird plumage and skin, feeding primarily on uropygial oil. While typically non-parasitic, they may cause skin irritation, especially in birds kept in captivity (Blanco *et al.,* 2001; Labrador *et al.,* 2022). Social birds are at greater risk acquiring mites due to close contact with conspecifics. In fact, group-living passerines are more likely to harbour feather mites than solitary species (Poulin 1991). Similarly, seasonal variations in feather mite infestations between migratory and resident birds have been reported (Galván *et al.,* 2008).

Here, we present the first survey of ectoparasites, including ticks, louse flies, chewing lice, and feather mites in wild birds from Menorca (Balearic Islands). Our primary objective is to describe the ectoparasite and feather mite communities present in this insular system and establish baseline host–parasite associations. In addition, we explore whether prevalence differs between migratory and sedentary species, as well as across seasonal sampling periods, to provide insights into the ecological factors shaping ectoparasite distributions.

## Material and methods

### Field sampling

Fieldwork was carried out in four locations of Menorca Island (Balearic Islands, Spain): (a) Illa de s’Aire (39° 48′ 02″ N, 4° 17′ 25″ E), a natural area that consists of an uninhabited islet (34 ha) located in the south-eastern part of Menorca; two natural areas within inland Menorca, (b) Alfurí de Dalt, (40º 02′ 14″ N, 3º 58′ 42″ E), (c) Albufera des Grau (39° 58′ 35″ N, 4° 14′ 23″ E) and d) the recovery centre for autonomous wild animals of Menorca (CRFSM) (40º 00′ 16″ N, 3º 52′ 03″ E), a facility for injured wildlife. Sampling on Illa de s’Aire was conducted during the prenuptial (30 April–1 May 2022) and postnuptial (4 October 2022) bird migration periods. Sampling on inland sites took place on eight occasions between 8 May and 31 July 2022. Birds were captured using mist nets during regular ringing campaigns by the Society of Ornithologists of Menorca (SOM). All selected sites are well known stopovers during bird migration. The captured birds were identified to species level using identification keys (Svensson *et al.,* 2010) and Avibase (https://avibase.bsc-eoc.org). We also broadly classify the bird species into sedentary (year-round residents in Menorca) or migratory (long-distance migrants) based on Svensson (2010) and ‘Estatus de l’avifauna Balear’ (López-Jurado, 2022). Those species that could belong to both migratory and sedentary populations, were considered as migratory or sedentary based on regional distribution and phenological data. Birds were examined for ectoparasites (ticks, louse flies and chewing lice) and feather mites by visual inspection of the head and body plumage, primary and secondary wing feathers, and tail feathers. Each bird was inspected for approximately 5–10 min to minimize handling time and stress. Inspections were always conducted by at least two people. Birds were placed over a white paper to facilitate the detection of any dislodged parasites. In addition, wings were examined against the light to enhance the visibility of feather mites. A 10× magnifier lens were also used to magnify and confirm the presence of small ectoparasites. The duration of inspection was kept consistent across individuals, independent of their size, health, or stress level. Ectoparasites were extracted using tweezers, brushes, or a swab and stored in 1.5 mL Eppendorf tubes containing 96% ethanol for further morphological identification. The presence of ectoparasites in birds at the CRFSM was assessed upon their arrival by the centre’s staff following the same protocol between February and November 2022.

### Morphological identification of ectoparasites and mites

Ticks (Ixodidae) were identified to species level (when possible) following the keys by Krantz and Walter (2009) and Estrada-Peña *et al.* (2017). Louse flies (Hippoboscidae) were identified using standard taxonomic keys (Hutson, 1984). Chewing lice (Phthiraptera) and feather mites (Astigmata) were mounted on Hoyer’s medium on microslides and sealed with Glpt insulating varnish. Lice were identified to the species level using specific taxonomic keys (Martín Mateo, 2002, 2009), except for the two newly recorded species in Spain. To identify these species, host associations were first confirmed following Price *et al.* (2003), and subsequently verified using the descriptions, as no dichotomous keys currently exist for these taxa. *Ardeicola expadillus* was identified based on the original description by Blagoveshtchensky (1940); nevertheless, this identification remains tentative and awaits a formal redescription of the species. For the identification of *Kurodaia haliaeeti*, the description provided by Price and Beer (1963) was used. Feather mites were identified to species level under optical microscopy following specific literature (Atyeo and Braasch, 1966; Karg, 1971; Kolarova and Mmitov, 2008; Kolarova, 2021).

Voucher specimens of lice, mites, ticks, and louse flies were deposited at the laboratory of the Applied Zoology and Animal Conservation research group, University of Balearic Islands, Spain.

### Statistical analysis

We compared the prevalence (presence or absence) of ectoparasites and feather mites between sedentary and migratory birds using Fisher test. Differences in ectoparasite prevalence were assessed by ticks, louse flies, and lice together and independently due their different behaviour.

Additionally, we tested for differences in feather mite prevalence among migratory birds of the four species captured in both migration periods in Illa de s’Aire (*Erithacus rubecula, Ficedula hypoleuca, Phoenicurus phoenicurus*, and *Phylloscopus trochilus*) during pre- (May) and postnuptial (October) migration. Only birds captured in Illa de s’Aire and inland Menorca sites were included in the analyses, while samples from CRFSM were excluded since these individuals were usually injured or stressed.

All analyses were performed using R version 4.3.2 (R Core Development Team, 2016). A significance level of ≤0.05 was used to determine statistical significance.

## Results

**Figure 1.**
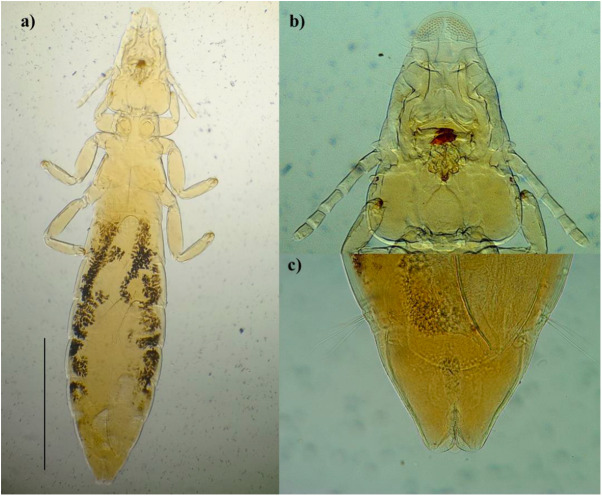
*Ardeicola expallidus.* (a) female body, (b) head and (c) terminal tergites of the abdomen. Scale: 1 mm.

**Figure 2.**
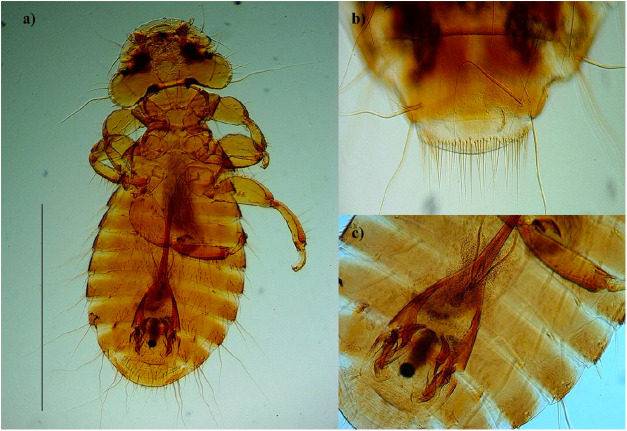
*Kurodaia haliaeeti* (a) male body, (b) terminal tergites of the abdomen from a female and (c) genitalia. Scale: 1 mm.

A total of 344 wild birds from 44 species were examined for ectoparasites and feather mites. Ectoparasites were detected in 70 birds (20.4%), comprising 18 different taxa: 13 species of lice, two of ticks, two of louse flies and one of mites. Among these, *Ardeicola expallidus* (Blagoveshtchensky, 1940) (Figure 1), and *Kurodaia haliaeeti* (Denny, 1842) (Figure 2), represent new records for the chewing louse fauna of Spain, while ten additional lice species are recorded here for the first time in the Balearic Islands (Table 1). Notably, the association between the lice *Ciconiphilus decimfasciatus* (Boisduval and Lacordaire, 1835) and the grey heron *Ardea cinerea* (Linnaeus, 1758) constitutes the first host–parasite record for Spain.

**Table 1.** Chewing lice on birds analysed in the CRFSM

Suborder	Species	Host bird (# of individuals)	Previous records in Spain	Reference
Amblycera	*Ciconiphilus decimfasciatus*	*Ardea cinerea* (1) ***	*Ardea purpurea, Bubulcus ibis, Egretta garzetta, Nycticorax nycticorax*.	(Martín Mateo 2002; Millán *et al.,* 2008)
		*Bubulcus ibis* (1)		
	*Dennyus hirundinis*	*Apus apus* (1)	*Apus apus, A. pallidus, Tachymarptis melva*	(Martín Mateo 2002)
	Colpocephalum percnopteri^**^	*Neophron percnopterus* (1)	*Neophron percnopterus*	(Martín Mateo 2002; Price and Beer 1963)
	Kurodaia haliaeeti^*^	*Pandion haliaetus* (1)	–	(Price and Beer 1963)
	Pseudomenopon pilosum^**^	*Fulica atra* (1)	*Fulica atra*	(Martín Mateo 2002)
	Laemobothrion maximum^**^	*Milvus milvus* (1)	*Aquila chrysaetus, Buteo buteo, Circus aeruginosus, Gyps fulvus, Milvus migrans, Milvus milvus, Pernis apivorus*	(Martín Mateo 2002)
	Laemobothrion tinnunculi^**^	*Falco tinnunculus* (1)	*Buteo buteo, Falco naumanni, Falco tinnunculus, Gypaetus barbatus*	(Martín Mateo 2002; Pérez *et al.,* 1996; Talabante and Bernal 2023)
Ischnocera	Aegypoecus perspicuus^**^	*Neophron percnopterus* (1)	*Neophron percnopterus*	(Martín Mateo 2009)
	Ardeicola expallidus^*^	*Bubulcus ibis* (1)		(Blagoveshtchensky 1940)
	Degeeriella fusca^**^	*Circus aeruginosus* (3)	*Circus aeruginosus, C. cyaneus, C. pygargus*	(Martín Mateo 2009)
	Quadraceps punctatus^**^	*Larus michahellis* (1)	*Larus michahellis* and *Chroicocephalus ridibundus*	(Martín Mateo 2009)
	Saemundssonia lari^**^	*Larus audouinii* (1)	*L. argentatus, L. audouinii, C. ridibundus*	(Martín Mateo 2009)
		*Larus michahelis* (3)		
	*Upupicola upupae*	*Upupa epops* (2)	*Upupa epops*	(Mester 1977; Martín Mateo 2009)

* First record for Spain.

** First record for the Balearic Islands.

*** First interaction record in lice-host bird for Spain.

### Ectoparasites and feather mites in captured wild birds

Of the 312 will birds examined from Illa de s’Aire and inland Menorca, 42 individuals (13.5%) were parasitized by at least one ectoparasites or feather mites. A total of 149 birds were captured from Illa de s’Aire, including 13 individuals of six species of sedentary birds and 136 individuals of 18 species of migratory birds (Table 2). Among the migratory birds on Illa de s’Aire, 63 and 73 individuals were captured during their prenuptial and postnuptial migration, respectively (Table 2). Additionally, 163 birds were captured in two inland sites in Menorca, including 153 individuals of 12 sedentary species and ten individuals of three migratory species (Table 2). Among the 163 wild birds captured in inland Menorca, eight birds (4.9%) from seven different species showed ectoparasites (Table 2). No birds captured on Illa de s’Aire (*N* = 149) were parasitized by ectoparasites. The prevalence of ectoparasites did not differ significantly between sedentary and migratory birds (*P* = 0.29; odds ratio = 2.69; 95% CI: 0.47–27.69).

**Table 2.** List of bird species captured in Illa de s’Aire and inland Menorca with their associated ectoparasites (number in brackets indicate the number of birds with these ectoparasites)

			Individuals			
Host	Sedentary	Migratory	Illa de s’Aire	Inland Menorca	Total individuals	Ectoparasites
*Carduelis carduelis*	X		3	7	10	
*Cettia cetti*	X		0	9	9	
*Chloris chloris*	X		0	8	8	*Hyalomma* sp. nymph (1)
*Erithacus rubecula*		X	45	0	45	
*Ficedula hypoleuca*		X	9	0	9	
*Fringilla coelebs*	X		0	12	12	*Hyalomma* sp. nymph (1)
*Hippolais polyglotta*		X	1	0	1	
*Hirundo rustica*		X	1	0	1	
*Lanius senator*		X	1	0	1	
*Linaria cannabina*	X		2	3	5	
*Luscinia megarhynchos*		X	6	6	12	
*Luscinia svecica*		X	1	0	1	
*Muscicapa striata*		X	6	0	6	
*Oenanthe oenanthe*		X	1	0	1	
*Otus scops*	X		0	2	2	
*Parus major*	X		0	13	13	*Ornithoica turdi* (1)
*Passer domesticus*	X		0	1	1	
*Phoenicurus phoenicurus*		X	12	0	12	
*Phylloscopus collybita*		X	3	0	3	
*Phylloscopus sibilatrix*		X	3	0	3	
*Phylloscopus trochilus*		X	38	1	39	
*Regulus ignicapilla*	X		3	1	4	
*Curruca communis*		X	2	0	2	
*Streptopelia turtur*		X	1	3	4	*Ornithophila mettalica* (1); *Ornithoica turdi* (1)
*Sylvia atricapilla*	X		3	31	34	*Hyalomma* sp. nymph (1)
*Sylvia borin*		X	1	0	1	
*Curruca subalpica*		X	1	0	1	
*Curruca melanocephala*	X		1	62	63	*Ornithoica turdi* (1)
*Turdus merula*	X		0	4	4	*Hyalomma* sp. nymph (1)
*Turdus philomelos*		X	4	0	4	
*Upupa epops*	X		1	0	1	

Ticks were observed in four birds. All of them captured inland Menorca on sedentary birds. All were immature stages identified as *Hyalomma* sp. (Linnaeus, 1758) (Table 2).

Louse flies were found on four birds from inland Menorca, including sedentary (*N* = 2), and migratory birds (*N* = 2). Two species were identified: *Ornithoica turdi* (Olivier in Latreille, 1811) (*N* = 3) and *Ornithophila metallica* (Schiner, 1864) (*N* = 1) (Table 2). The prevalence of louse flies did not differ significantly between sedentary and migratory birds (*P* = 1; odds ratio = 0.88; 95% CI: 0.06–12.2).

No chewing lice were found on birds captured either inland in Menorca or on Illa de s’Aire. All lice were recorded from birds received at the wildlife recovery centre.

Feather mites were recorded in 34 birds (10.4 %) captured in Illa de s’Aire and inland Menorca. On Illa de s’Aire, 15.4% (23/149) of birds carried mites, whereas 6.7% (11/163) of inland birds presented feather mites. Among sedentary species, 11.4% (*N* = 19/166) harboured mites (13 species), while 10.3 % (*N* = 15/146) of migratory birds were infested (18 species) (Table 3). Two mite species were identified: *Proctophyllodes sylviae* (Gaud, 1957), was found in 26 birds from 14 species, and *Trouessartia bifurcata* (Trouessart, 1884) was found in eight birds from three species. No significant difference in overall mite prevalence was found between sedentary and migratory birds (*P* = 0.86; odds ratio = 1.13; 95% CI: 0.52–2.49). We did not find significant differences in mite prevalence between migratory birds from the four species (*Erithacus rubecula, Ficedula hypoleuca, Phoenicurus phoenicurus*, and *Phylloscopus trochilus*) captured in both pre- (5.1%; *N* = 2/39) and postnuptial (15.2 %; *N* = 10/66) migration periods in Illa de s’Aire (*P* = 0.2; odds ratio = 0.3; 95% CI: 0.03–1.55).

**Table 3.** Mites found on birds captured on Illa de s’Aire during the prenuptial and postnuptial migrations, and inland Menorca. Numbers in brackets indicate the number of birds carrying these mite species. Species names underlined are considered migratory

Sampling site	Migration	Bird species	*N*	Prevalence	Mite species
Illa de s’Aire	Prenuptial	*Carduelis carduelis*	1	0	
		*Erithacus rubecula*	2	0	
		*Ficedula hypoleuca*	3	0	
		*Hippolais polyglotta*	1	0	
		*Hirundo rustica*	1	0	
		*Lanius senator*	1	0	
		*Luscinia megarhynchos*	6	0	
		*Muscicapa striata*	6	1	*Proctophyllodes sylviae*
		*Phoenicurus phoenicurus*	3	0	
		*Phylloscopus collybita*	3	0	
		*Phylloscopus sibilatrix*	3	0	
		*Phylloscopus trochilus*	30	2	*Proctophyllodes sylviae*
		*Curruca subalpina*	1	0	
		*Curruca communis*	2	0	
		*Streptopelia turtur*	1	0	
	Postnuptial	*Carduelis carduelis*	2	1	*Proctophyllodes sylviae*
		*Erithacus rubecula*	43	5	*Proctophyllodes sylviae* (5)
					*Trouessartia* sp. (1)
		*Ficedula hypoleuca*	6	3	*Proctophyllodes sylviae*
		*Linaria cannabina*	2	2	*Proctophyllodes sylviae*
		*Luscinia svecica*	1	1	*Proctophyllodes sylviae* (1)
					*Trouessartia bifurcata* (1)
		*Oenanthe oenanthe*	1	0	
		*Phoenicurus phoenicurus*	9	1	*Proctophyllodes sylviae*
		*Phylloscopus trochilus*	8	1	*Proctophyllodes sylviae*
		*Regulus ignicapilla*	3	2	*Proctophyllodes sylviae*
		*Sylvia atricapilla*	3	3	*Proctophyllodes sylviae*
		*Sylvia borin*	1	0	
		*Curruca melanocephala*	1	0	
		*Turdus philomelos*	4	0	
		*Upupa epops*	1	1	*Proctophyllodes sylviae*
Inland Menorca	Prenuptial	*Carduelis carduelis*	7	0	
		*Cettia cetti*	9	0	
		*Chloris chloris*	8	0	
		*Fringilla coelebs*	12	0	
		*Linaria cannabina*	3	0	
		*Lusciniamegarhynchos*	6	1	*Proctophyllodes sylviae*
		*Otus scops*	2	0	
		*Parus major*	13	1	*Proctophyllodes sylviae*
		*Passer domesticus*	1	0	
		*Phylloscopus trochilus*	1	0	
		*Regulus ignicapilla*	1	0	
		*Streptopelia turtur*	3	0	
		*Sylvia atricapilla*	31	3	*Trouessartia bifurcata*
		*Curruca melanocephala*	62	6	*Proctophyllodes sylviae* (1)
					*Trouessartia bifurcata* (4)
					*Trouessartia* sp. (1)
		*Turdus merula*	4	0	

### Ectoparasites and feather mites in birds from the wildlife recovery centre

Among the 32 birds from 16 species examined at the CRFSM, 28 individuals (87.5%) from 14 species were parasitized by at least one type of ectoparasite (chewing lice, louse fly or tick) (Table 4). The louse fly *O. metallica* was found on the Eurasian hoopoe *Upupa epops* (Linnaeus, 1758). An adult *Hyalomma lusitanicum* (Koch 1844) was identified on a yellow-legged gull *Larus michahellis* (Naumann, 1840), and immature stage of *Hyalomma* sp. were found on two stone-curlews *Burhinus oedicnemus* (Linnaeus, 1758). The tick *Rhipicephalus sanguineus* s.l. (Latreille, 1806) was found on a red kite *Milvus milvus* (Linnaeus, 1758), and an immature stage of *Ixodes* sp. were found on a barn owl *Tyto alba* (Scopoli, 1769). A soft-bodied tick, *Ornithodorus maritimus* (Vermeil and Marguet, 1967) (Argasidae) was found on a cattle egret *Bubulcus ibis* (Linnaeus, 1758). Thirteen species of lice (Phthiraptera) were found on 67.7% (*N* = 21) of the CRFSM birds (Table 4). In addition to the new national and regional lice records described above, the mite *Parasitus fimetorum* (Berlese, 1904) was identified on *Larus michahellis*. The feather-mites *Ornithonyssus sylviarium* (Canestrini and Fanzago, 1877) was detected on the Eurasian scops owl *Otus scops* (Linnaeus, 1758)*, Ornithonyssus* sp. on a house sparrow *Passer domesticus* (Linnaeus, 1758) and Eurasian scops owl, and *Nothoapis* sp. on a cattle egret.

**Table 4.** Ectoparasites identified on the birds analysed in the CRFSM. The prevalence corresponds to the total number of ectoparasites collected from each host species

Bird species	Individuals	Ectoparasite group	Ectoparasite species	N	Prevalence
*Ardea cinerea*	1	Amblycera	*Ciconiphilus decimfasciatus*	6	6
*Apus apus*	1	Amblycera	*Dennyus hirundinis*	3	3
*Bubulcus ibis*	4	Amblycera	*Ciconiphilus decimfasciatus*	16	4
		Ischnocera	*Ardeicola expallidus*	5	1.25
		Argasidae	*Ornithodoros maritimus*	1	0.25
*Burhinus oedicnemus*	2	Ixodidae	*Hyalomma* sp. (nymph)	15	7.5
		Ixodidae	*Hyalomma* sp. (nymph)	6	3
*Circus aeruginosus*	3	Ischnocera	*Degeeriella fusca*	17	5.7
*Falco tinnunculus*	2	Amblycera	*Laemobothrion tinnunculi*	9	4.5
		Ischnocera	*Degeeriella fusca*	2	1
*Fulica atra*	1	Amblycera	*Pseudomenopon pilosum*	1	1
*Ichthyaetus audouinni*	1	Ischnocera	*Saemundssonia* cf*. lari*	5	5
*Larus michahellis*	4	Ischnocera	*Saemundssonia* cf. *lari*	19	4.75
		Ischnocera	*Quadraceps punctatus*	1	0.25
		Ixodidae	*Hyalomma lusitanicum*	1	0.25
*Milvus milvus*	2	Amblycera	*Laemobothrion maximun*	1	0.5
		Ixodidae	*Rhipicephalus sanguineus* s.l.	3	1.5
*Neophron percnopterus*	1	Amblycera	*Colpocephalum percnopteri*	2	2
		Ischnocera	*Aegypoecus perspicuus*	20	20
*Pandion haliateus*	1	Amblycera	*Kurodaia haliaeeti*	11	11
*Tyto alba*	1	Ixodidae	*Ixodes* sp.	1	1
*Upupa epops*	4	Ischnocera	*Upupicola upupae*	27	6.75
		Hippoboscidae	*Ornithophila metallica*	1	0.25

## Discussion

This study provides the first comprehensive analysis of ectoparasites and feather mites in wild sedentary and migrant passerines from Menorca (Balearic Islands). We reported 13 species of chewing lice, two of louse flies, two of ticks, and one of mites, with *A. expallidus* and *K. haliaeeti* as new records for Spain, and ten lice species newly recorded from the Balearic Islands. Most of these new records originated from birds admitted to the wildlife recovery centre (CRFSM). These findings represent interesting geographical records but not new host–parasite associations, as all lice species had been previously reported from the same or closely related host species. *Ardeicola expallidus* has been previously recorded on birds of the Ardeidae family, such as cattle egret, great egret *Ardea alba* (Linnaeus, 1758), and snowy egret *Egretta thula* (Molina, 1782) (Price *et al.,* 2003). This ectoparasite was also recorded in other European countries like Bulgaria (Balát 1958; Ilieva, 2009, Touleshkov 1974), Slovakia (Balát, 1953, 1956, 1977), Hungary (Rékási, 2002), Faroae Islands (Palma and Jensen, 2005) and Romania in great egret and the little egret *Egretta garzetta* (Linnaeus 1766) (Rékási *et al.,* 2017). The other new record for Spain is *K. haliaeeti*, which exclusively parasitizes Ospreys (Price *et al.,* 2003). Our study confirms the high specificity of most of the species sampled. The different species of lice found parasitizing birds in this study have been previously found on the same bird species, including *C. decimfasciatus* on grey heron (Touleshkov, 1958, 1974; Palma and Jensen, 2005; Gustafsson *et al.,* 2019; Vas *et al.,* 2012). This lice species was previously recorded in Spain on a bird species of the same genus: the purple heron *Ardea purpurea* (Linnaeus, 1766) (Millán *et al.,* 2008).

The high prevalence of ectoparasites observed in birds at the CRFSM likely reflects their compromised health status. Similar findings were reported in Portugal, where stressed or injured birds had higher ectoparasite loads (Tomás *et al.,* 2016). Many of the birds examined at the CRFSM were debilitated, which could have increased their susceptibility to infestation. Indeed, captive conditions can influence ectoparasite dynamics through behavioural changes in birds. For example, stress or confinement can temporarily reduce grooming, facilitating parasite proliferation, although this effect likely only appears after several parasite generations. In contrast, birds kept in captivity for longer periods may groom more frequently than wild individuals, as they spend less time foraging or monitoring for predators (Waite *et al.,* 2012; Villa *et al.,* 2019; Bush *et al.,* 2023). However, since ectoparasites were collected upon arrival at the recovery centre, before the birds’ shared enclosures, infestation due to the factors previously described and/or cross-infection within the centre is unlikely. Only occasional accidental transfer, particularly of feather mites, during transport would be possible.

Previous studies in Europe have reported high rates of ectoparasite infections in Accipitriformes, with a lice prevalence of 41.8% in Spanish raptors (Pérez *et al.,* 1996) and 42.6 % in Turkish raptors (Inci *et al.,* 2010), or Eleonora falcons *Falco eleonorae* Géné, 1839 from Alegranza island also show high prevalence of louse flies (Gangoso *et al.,* 2019). Birds collected in the current study were mainly passerines (except in the CRFSM, where the birds inspected were birds of prey). According to Rozsa (1997), larger-bodied bird species tend to harbour more ectoparasites than smaller passerines. This is not only because of their greater surface area but also due to the relative size of the parasites. For example, a tick on a small passerine’s head is highly exposed and more likely to be removed during preening, whereas on a large raptor, it may remain unnoticed. Additionally, longevity could influence ectoparasite accumulation, as longer-lived species may provide more opportunities for colonization over time. However, the role of migration in shaping ectoparasite prevalence appears to differ among bird groups: in some non-passerines, host migration seems to have little effect on louse prevalence (Grossi *et al.,* 2023), whereas in passerines, higher ectoparasite loads in migratory species can have long-term fitness consequences, potentially reducing annual survival probability (Klaus *et al.,* 2016). This fact can greatly impact the return rate of parasite-infested birds from breeding sites to wintering grounds. Heavily infested birds often deplete their fat reserves to support an increased metabolic rate, leading to reduced body mass and lower survival rates, especially during migration (Brown and Sherry, 2006). The complete absence of ectoparasites in birds from Illa de s’Aire was striking. However, similar results were found by Gustafsson *et al.* (2019), which found a low parasitized rate by lice in migratory passerine birds. Given the relatively homogeneous climate across Menorca, this pattern likely reflects differences in the bird community rather than environmental conditions. High ectoparasite loads can increase energetic costs, reduce flight efficiency, and lower survival during migration. Consequently, heavily parasitized birds may fail to reach stopover sites such as Illa de s’Aire. This ‘migratory culling’ effect could therefore explain the complete absence of ectoparasites in the individuals captured on the islet (Satterfield *et al.,* 2018; Gangoso *et al.,* 2024).

The louse fly *O. turdi* recorded on *Curruca melanocephala, Streptopelia turtur*, and *Parus major*, has been documented previously in various passerine families, indicating low host specificity (Gaponov and Tewelde, 2020; Zittra *et al.,* 2020). While this species is primarily distributed in Africa and southern Europe (Oboňa *et al.,* 2019; González *et al.,* 2023), it has been also recorded across central Europe (Droz and Haenni, 2011). Its distribution is of special interest due to its potential role as vector for trypanosome parasites (Santolíková *et al.,* 2022). Keve *et al.* (2024) observed *O. turdi* more frequently on short-distance migrants, but we did not record significant differences in prevalence between migratory and sedentary birds, probably due to the low prevalence of louse flies in our sampled birds The other louse fly observed, *O. metallica*, found on *S. turtur* is a well-documented species that parasitize numerous bird species across Europe (Nartshuk and Matyukhin, 2019; Lehikoinen *et al.,* 2021). Although *O. metallica* predominantly parasitize sedentary birds, we found it on a migratory species. Further studies are needed to assess its potential role as vector for trypanosome parasites.

The prevalence of ticks observed in our study aligns with previous studies, where only a small number of migratory birds were usually parasitized by ticks (Elfving *et al.,* 2010; Jameson *et al.,* 2012; Klaus *et al.,* 2016; Espí *et al.,* 2023). For example, Espí *et al.* (2023), reported that only 2.5% of wild birds captured in Northwestern coastal Spain were parasitized by ticks. While we observed a marginally higher tick prevalence in sedentary birds than in migratory ones, the difference was not statistically significant, likely due to the low number of positive cases. In contrast, Klaus *et al.* (2016), in Germany, reported significantly higher tick infestations in sedentary birds compared to migratory ones. The prevalence of ticks on different species depends mainly on the degree of feeding on the ground (Hasle, 2013). Ground-feeding species, such as the common blackbird (*Turdus merula*), often shows higher tick prevalence than non-ground feeders (Klaus *et al.,* 2016). In fact, in our study, 25% of the common blackbirds analysed were parasitized by ticks. Ticks collected from birds in our study were predominantly identified as *Hyalomma* sp., consistent with previous reports where *Hyalomma* ticks have been frequently associated with migratory birds (Capek *et al.,* 2014; Estrada-Peña *et al.,* 2021). In particular, *Hyalomma marginatum* is commonly transported into Europe along migratory routes and is a known vector of Crimean-Congo haemorrhagic fever virus (CCHFV; Lindeborg *et al.,* 2012). Although CCHFV detection was beyond the scope of this study, the presence of *Hyalomma* sp. on migratory birds underlines the potential risk for the introduction and spread of zoonotic pathogens in the study area.

Regarding feather mites, the two species of mites found in our study have been previously reported in Spain at high prevalence levels on Eurasian blackcap *Sylvia atricapilla* (Linnaeus, 1758) (Fernández-González *et al.,* 2013). These mites often co-occur on Eurasian blackcap (Mironov *et al.,* 2012). However, they have also been reported on other bird species from various families. For example, *P. sylviae*, has been documented on the common reed warbler *Acrocephalus scirpaceus* (Hermann, 1804), the Cetti’s warbler *Cettia cetti* (Temminck, 1820), the common chiffchaff *Phylloscopus collybita* (Vieillot, 1817) among others from several regions of Eurasia (Mironov, 1996; Per and Aktaş, 2018). Similarly, *T. bifurcata* has been observed on great reed warbler *Acrocephalus arundinaceus* (Linnaeus, 1758), Cetti’s warbler (Per and Aktaş, 2018), and greater whitethroat *Curruca communis* (Latham, 1787) (Siepel *et al.,* 2023). These previous studies confirm our results, where we found *P. sylviae* in 14 different species from eight families and *T. bifurcata* on three species from two families.

Although Marčanová and Janiga (2021) found that mite prevalence reached its maximum after the bird’s postnuptial moult, suggesting that mites preferred newly coated feathers after a moult, when we analysed only migratory birds, we failed to find differences in the prevalence of feather mites between those individuals captured during prenuptial migration and postnuptial migration. However, our result could be due to the highly unbalanced counts between periods (Table 3). Therefore, further studies are required to assess within-species seasonal changes in feather mite prevalence.

The current study provides the first comprehensive overview of the ectoparasite community in wild sedentary and migratory passerines from Menorca. The detection two new chewing lice records for Spain (*A. expallidus* and *K. haliaeeti*) and ten lice species newly recorded for the Balearic Islands, highlights the still underexplored diversity of avian ectoparasites in the Mediterranean region. Although no significant differences in prevalence were found between sedentary and migratory birds, these findings emphasize the importance of continuous monitoring and the use of complementary sampling methods to improve ectoparasite detection. Further studies integrating pathogen screening are needed to better understand the ecological and epidemiological implications of these host–parasite associations on Mediterranean islands.

## Data Availability

The data presented in this study are available on request from the corresponding author.
